# Urachus adenocarcinoma mistaken for umbilical incision implant cancer after laparoscopic cholecystectomy: a case report

**DOI:** 10.3389/pore.2023.1611334

**Published:** 2023-12-22

**Authors:** Yanxing Mai, Lei Feng, Zhenxi Liu, Yu Nie, Zesheng Jiang, Jiasheng Qin

**Affiliations:** ^1^ Department of Geriatrics, Zhujiang Hospital, Southern Medical University, Guangzhou, China; ^2^ Department of Hepatobiliary Surgery II, General Surgery Center, Guangdong Provincial Research Center for Artificial Organ and Tissue Engineering, Guangzhou Clinical Research and Transformation Center for Artificial Liver, Institute of Regenerative Medicine, Zhujiang Hospital, Southern Medical University, Guangzhou, China; ^3^ Department of Hepatobiliary Surgery, The Affiliated Hospital of Guizhou Medical University, Guiyang, China; ^4^ Department of Pathology, Zhujiang Hospital, Southern Medical University, Guangzhou, China

**Keywords:** urachus adenocarcinoma, laparoscopic cholecystectomy, umbilical incision implant cancer, invasive adenocarcinoma, case report

## Abstract

Umbilical incision implant cancer after LC is rare. Elective cholecystectomy was planned for a 49 years-old female patient with symptomatic gallstones. The patient underwent transumbilical single-port LC after admission to our hospital. Gallbladder specimens were obtained directly through the umbilical puncture hole, and histopathology suggested chronic cholecystitis. Three months after surgery, the patient experienced painful induration in the umbilicus. We initially considered incision scar hyperplasia complicated with pain, and used drugs to treat it conservatively without taking special treatment measures. Six months after LC, the umbilical induration pain affected her quality of life, and the patient requested surgical resection. Preoperative ultrasonography and abdominal computerized tomography (CT) revealed nodular changes around the umbilicus and no abdominal mass. Local resection of the periumbilical mass was performed, and the pathological confirmation was invasive adenocarcinoma. Subsequently, the patient underwent repeat periumbilical mass enlargement resection. Postoperative pathology showed no cancer at the enlarged resection margin, yet the umbilical center pathology showed invasive adenocarcinoma. The excised pathology was sent to the Sun Yat-sen University Cancer Center for consultation because of the rare nature of the findings associated with the case. After consultation, a diagnosis of umbilical urachus adenocarcinoma was confirmed based on pathological morphology, immunohistochemistry, and the specific anatomical location of the tumor. This case report shown that when there is a persistent mass induration in the navel after LC surgery, the possibility of incision tumor should be considered, rather than simply excluding the possibility of a cancer based on a non-cancer medical history.

## Introduction

Transumbilical laparoscopic surgery is widely practiced, and cases of poor healing of umbilical incisions after surgery are often encountered in the clinic. Incisional metastases from internal malignancies are uncommon [1]. The urachus is an embryonic remnant of the urogenital sinus and allantois [2], which is used as a channel connecting the fetus and mother. Following embryogenesis, the urachus normally obliterates into the umbilical ligament; however, occasionally it does not undergo complete atresia [3]. There is a catheter in the umbilical cord that communicates with the bladder, and an urachus fistula is formed after laparoscopic transumbilical access, which is also occasionally encountered in the clinic. Most urachus adenocarcinomas occur when the end of the umbilical urethra meets the top of the bladder and is hidden [4]. Here, we report a rare case of a patient with chronic calculous cholecystitis whose cholecystectomy specimens were histologically non-cancerous after laparoscopic cholecystectomy (LC) surgery. However, following LC surgery, the patient developed painful induration in the navel, which was finally diagnosed as urachus adenocarcinoma in the umbilicus 3 months after LC surgery.

## Case report

**FIGURE 1:**
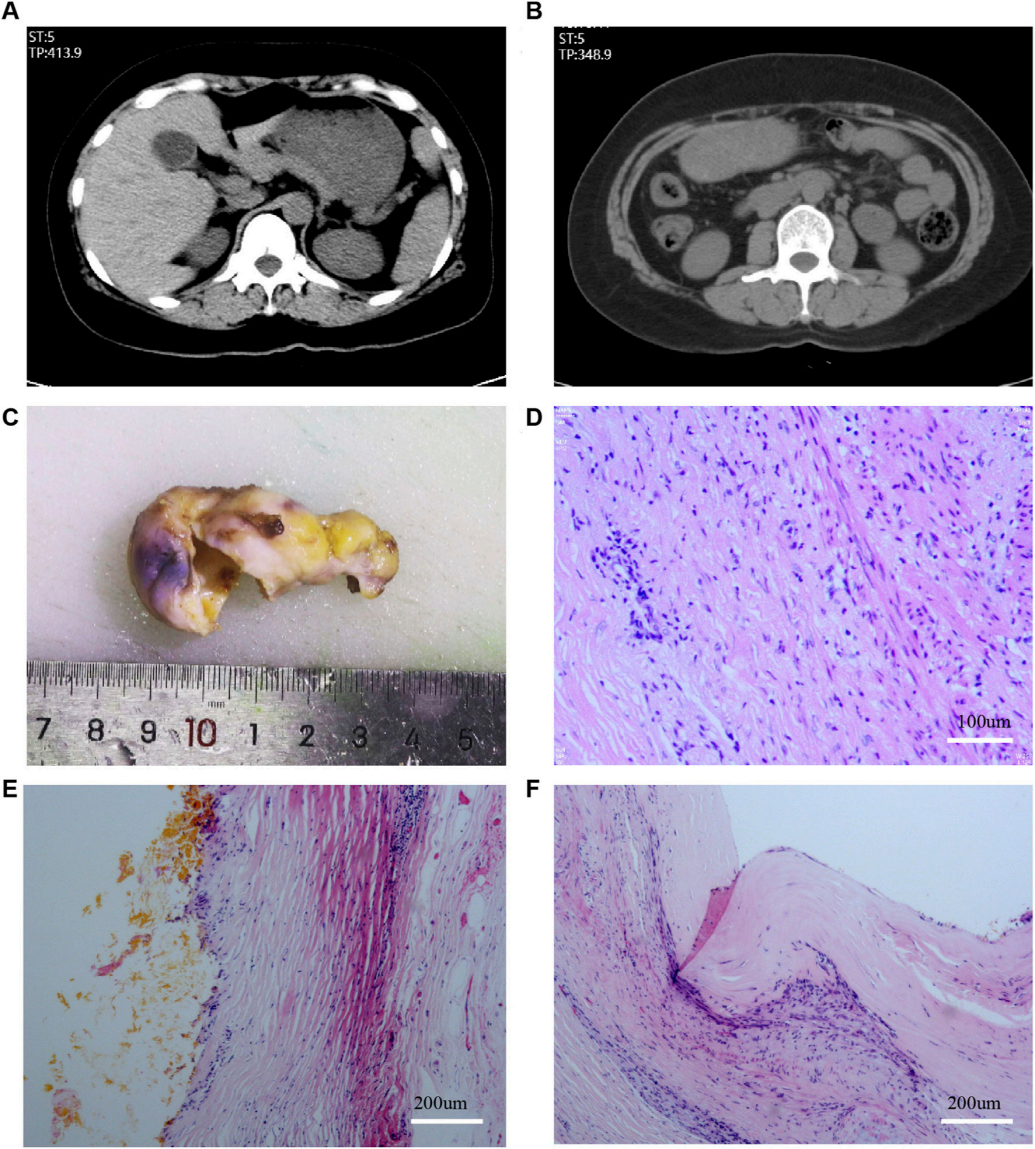
Plain scan CT examination before LC and pathology after LC. **(A,B)**: CT examination before LC; **(C)**: Gross specimen of the gallbladder; **(D–F)**: Results of HE staining of the gallbladder.

This was a case of urachus adenocarcinoma after laparoscopic cholecystectomy, confirmed by pathological consultation at Zhujiang Hospital and Sun Yat-sen University Cancer Center. Clinical data, including clinical symptoms, signs, radiological findings, laboratory analyses, pathological diagnosis, and treatment strategies, were obtained from the hospital’s electronic medical records. Pathological diagnoses, including immunohistochemistry (IHC), were independently reviewed by two pathologists. The patient and her family approved the anonymous use of the data in accordance with the Helsinki Declaration. Elective cholecystectomy was planned for a 49 years-old female patient with symptomatic gallstones. A computerized tomography (CT) scan showed a gallbladder wall thickness of 4 mm, and no positive stones (Figures 1A, B). Therefore, gallstones with chronic cholecystitis were diagnosed.

The patient underwent transumbilical single-port LC after admission to our hospital in August 2021, and no obvious lesions were observed in the abdominal cavity. The gallbladder specimen was obtained directly through the umbilical puncture hole without using Endobag or other specimen bag tools. The incision diameter was approximately 1.5 cm, and was closed using Johnson & Johnson 2-0 and 4-0 absorbable Vicjo lines (Coated Vicryl Plus Antibacterial Suture). No gallbladder rupture or bile extravasation occurred during the operation, and no drainage tube was placed. The patient recovered well after the operation, was discharged the following day, and the abdominal incision healed normally. Histopathology suggested chronic cholecystitis, and no pathological manifestations of accidental gallbladder cancer or dysplasia were reported (Figures 1C–F).

Three months after LC surgery, the patient found that her umbilical subcutaneous nodule was hard, with noticeable pain, which worsened after activity; however, there was no abnormality in appearance. During the outpatient follow-up incision scar hyperplasia and pain were reported, and conservative treatment was provided with no special treatment measures taken.

**FIGURE 2:**
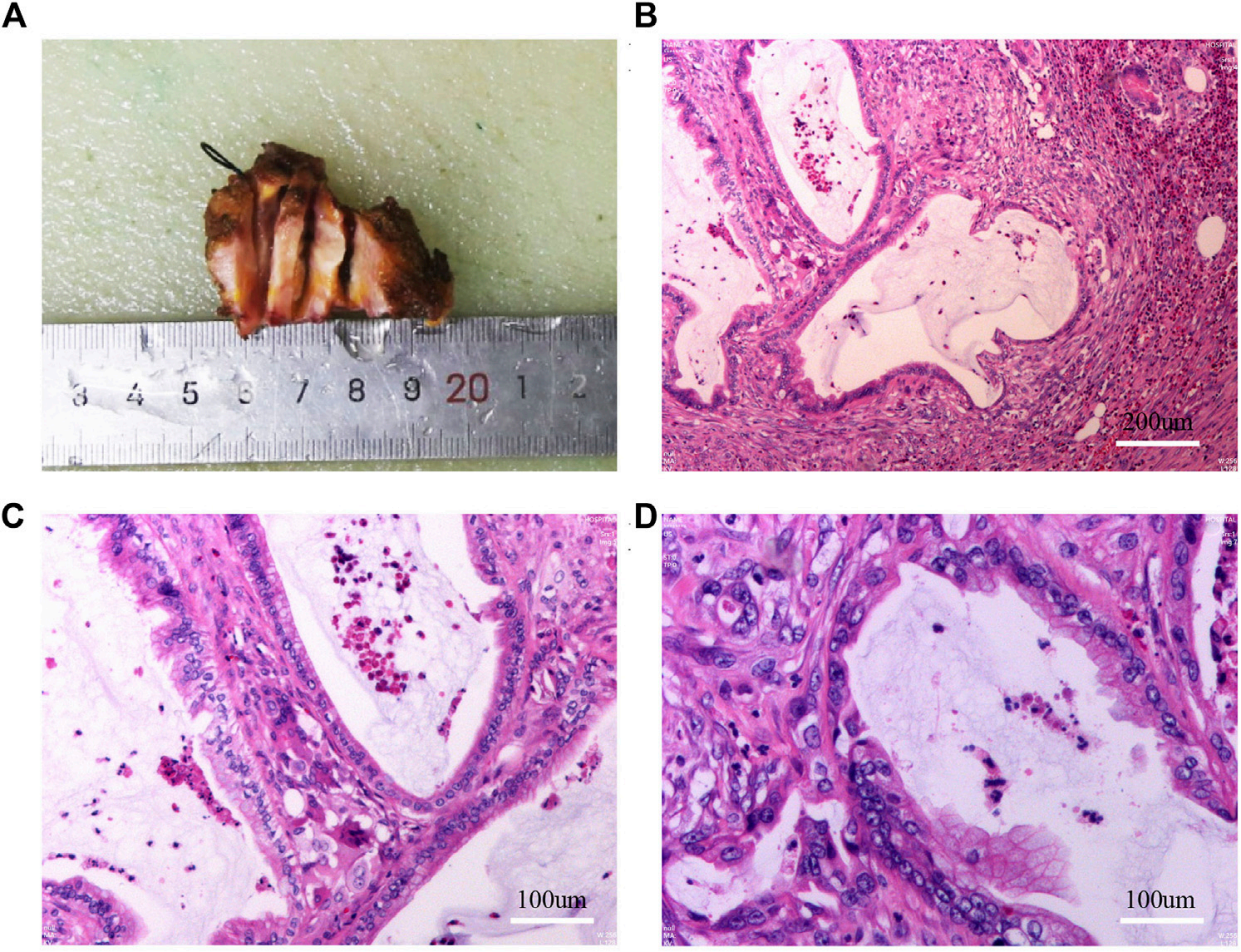
Results of gross specimen, HE staining after the first umbilical nodule resection. **(A)**: Gross specimen after the first umbilical nodule resection; **(B–D)**: HE staining after the first umbilical nodule resection.

In April 2022, because the pain in the umbilical nodules was affecting her quality of life, the patient requested surgical resection. Preoperative abdominal wall ultrasound showed uneven hypoechoic nodules under the skin of the navel, approximately 17 × 11 mm in size (Supplementary Figures S1A, B). The boundary was visible, no capsule was seen, and color Doppler flow imaging showed no blood flow signal. Abdominal plain CT scan showed subcutaneous nodules in the umbilical region with uniform density, approximately 2.2 cm × 3.1 cm in size (Supplementary Figures S1C, D). After the multidisciplinary team discussion, we made the following preoperative potential diagnoses: 1) suture reaction, 2) desmoid tumor, 3) gastrointestinal or gynecological malignancy, 4) scar hyperplasia with pain, 5) postoperative adhesions of the abdominal wall, 6) traumatic neuroma, 7) accidental gallbladder cancer implantation metastases. We then performed surgery by inserting the incision 10 cm to the left side of the original umbilical incision. No abdominal adhesions were found in laparoscopic exploration. However, a white hard mass in the umbilical cord was visible (Supplementary Figures S1E, F), and umbilical nodule resection was performed during the operation. Invasive adenocarcinoma was diagnosed postoperatively, and IHC analysis identified the following cancer cells: CK (+), CK20(+), CEA (+), CDX-2(−), CR part (+), TIF-1(−), CA199(+), SYN(−), CgA(−), D2-40(−), MC(−), WT-1(−), KI-67 hotspot about 30% (+) (Figures 2A–D). We first considered the source to be the digestive system because of the IHC results and the patient’s medical history, but did not exclude the possibility of cholangiocyte origin. The pathological findings suggested that the adenocarcinoma had no specific direction, so it was recommended to clinically exclude the possibility of secondary metastasis and the tumor at the primary site needed to be eliminated. Thus, we first considered whether the patient had not shown a positive result on the routine pathological examination due to accidental miss of gallbladder cancer. Surprisingly, despite repeated histological examinations and independent reviews by multiple pathologists in the archival gallbladder tissue section, no primary cancer was diagnosed, the diagnosis of chronic cholecystitis was confirmed, and no pathological changes in dysmorphic cells and cell dysplasia were observed.

**FIGURE 3:**
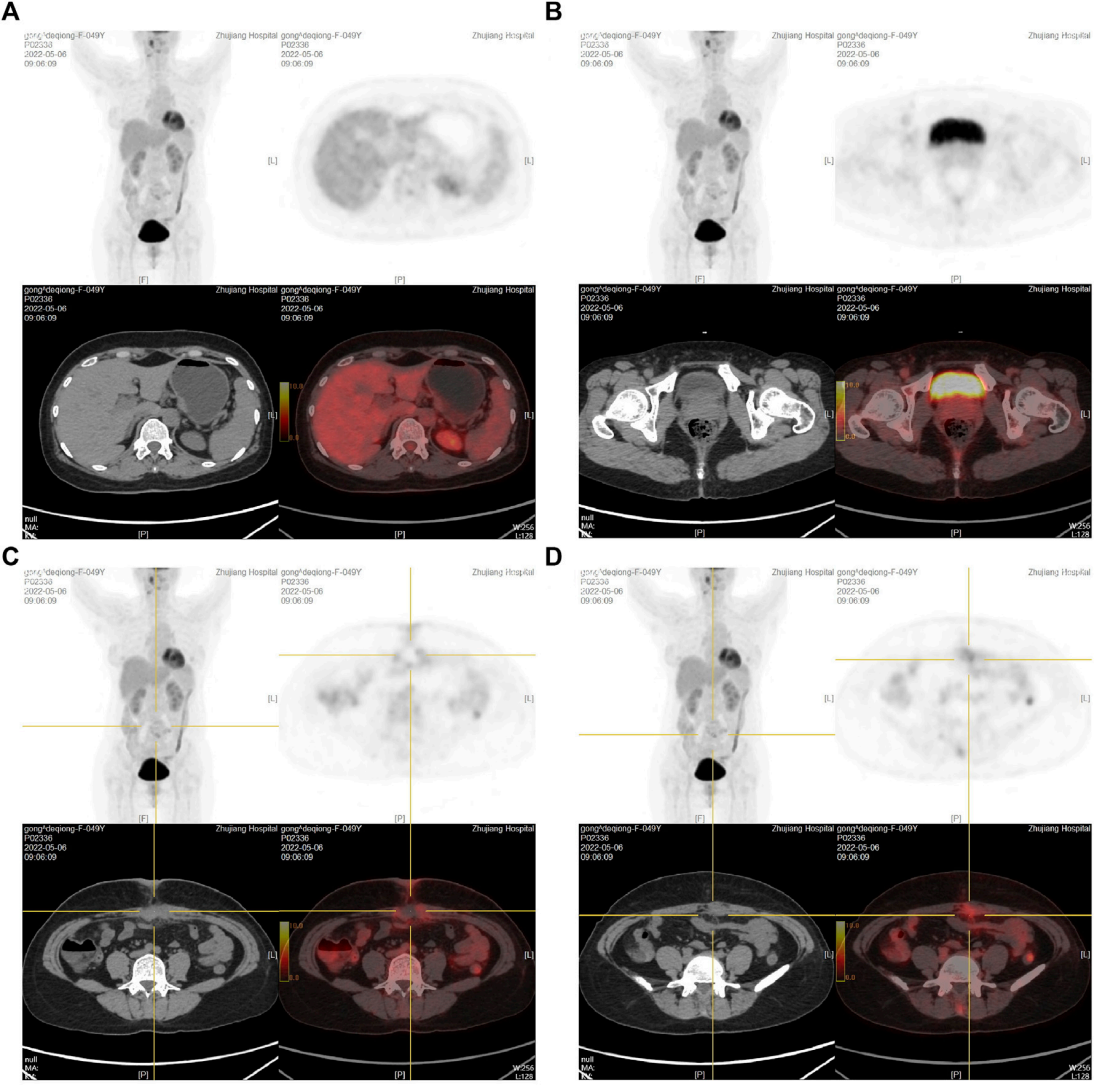
PET-CT examination after the first umbilical nodule resection. **(A)**: PET-CT results at the gallbladder fossa level; **(B)**: PET-CT results at the bladder level; **(C,D)**: PET-CT results at the umbilical nodule level.

**FIGURE 4:**
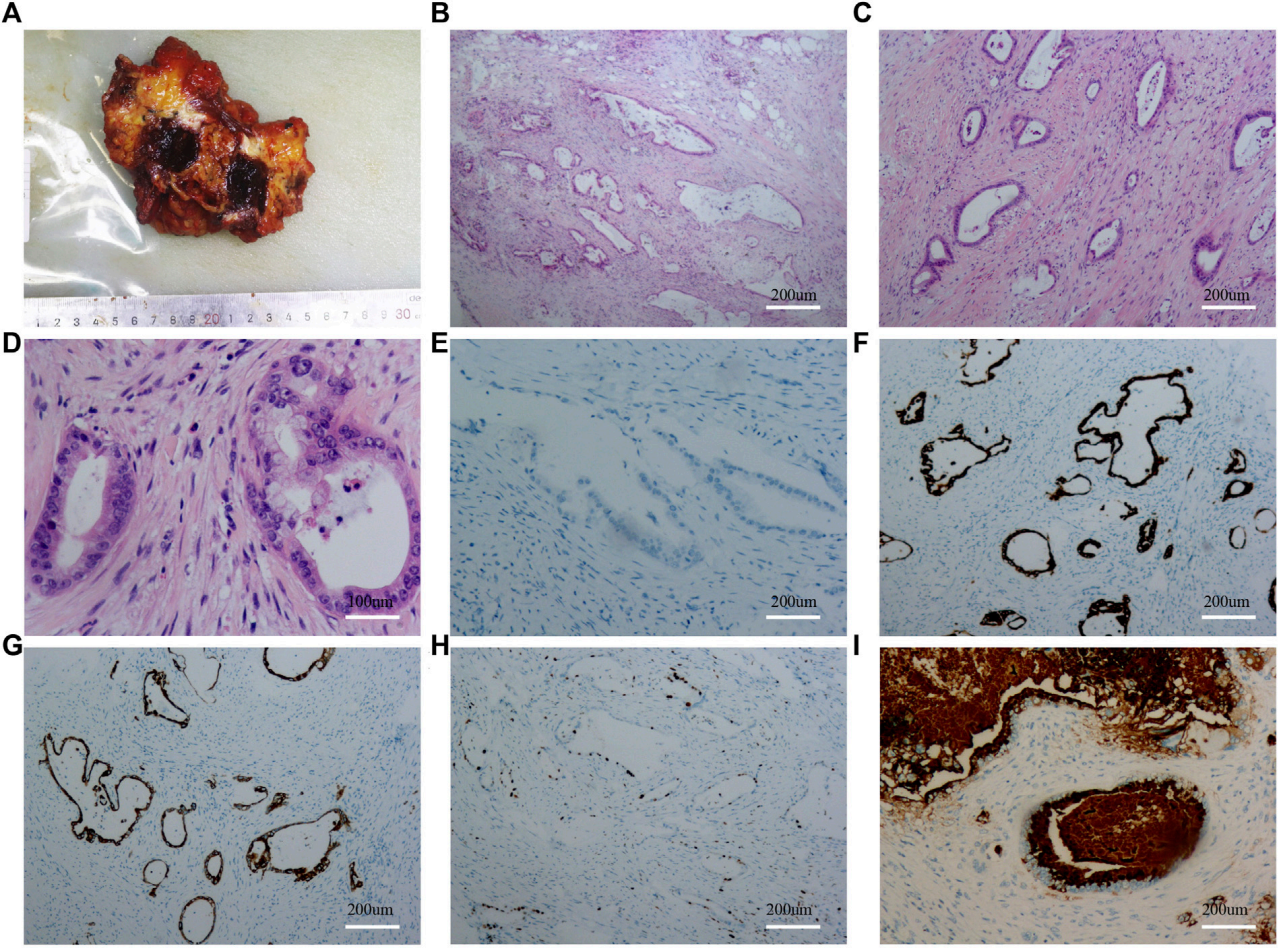
Results of gross specimen, HE staining and immunohistochemistry after the second umbilical nodule resection. **(A)**: Gross specimen after the second umbilical nodule resection; **(B–D)**: HE staining after the second umbilical nodule resection; **(E)**: CDX-2 staining after the second umbilical nodule resection; **(F)**: CK7 staining after the second umbilical nodule resection; **(G)**: CD20 staining after the second umbilical nodule resection; **(H)**: KI67 staining after the second umbilical nodule resection; **(I)**: MUC5AC staining after the second umbilical nodule resection.

To clarify the possible primary lesion of the mass, the patient underwent upper abdominal enhanced CT and systemic positron emission tomography (PET)-CT examination; however, no systemic lesion imaging findings were found in the rest of the body, including intra-abdominal tumors, except for the umbilical lesions (Supplementary Figures S2A, B and Figures 3A–D). Moreover, there were no positive findings on gastroscopy or colonoscopy (Supplementary Figures S2C, D). We then immediately performed a periumbilical mass enlargement resection, including approximately 5 cm of normal tissue around the tumor. A mesh was placed to repair the resection wound, because of the large size of the peritoneal defect. Postoperative histopathology showed that moderately differentiated invasive adenocarcinoma tissue could still be detected in the umbilical center, and there was no clear invasion of the vasculature and nerves. IHC analysis revealed the following: MUC-5AC(+), MUC-6 (focal +), MUC-2 (−), P16 (focal +), CK7 (+), CK20(+), CDX-2 (−), P53 (20%, wild-type expression), Ki-67 (approximately 35%+), MLH1 (+), MSH2(+), MSH6(+), and PMS2(+). In addition, cancerous tissue was not detected in the 3-6-9-12 points of the specimen’s margins (Figures 4A–I).

Because of the rarity of the findings associated with this case, both the umbilical nodule resection and the second enlarged resection specimens were sent to the Sun Yat-sen University Cancer Center for consultation. Consultation results suggested high to moderately differentiated adenocarcinoma invasion, with the following IHC findings: CK20(+), CDX-2 (individual weak+), SATB2(−), CK7(+), CEA (+), and HER-2(0). The diagnosis was finally determined to be urachus adenocarcinoma, based on the tumor morphology combined with the IHC results and its specific location, thus excluding secondary metastatic cancer. The patient recovered well after surgery and was discharged from the hospital after the incision healed. In this specific clinical practice, the original pathology indicated invasive adenocarcinoma and the patient’s surgical history suggested potential gallbladder cancer; therefore radiotherapy was used. However, the patient only underwent tumor localization for radiotherapy planning and the first dose of radiotherapy. As the results obtained from the subsequent pathological consultation suggested urachal carcinoma, sensitivity to radiotherapy was considered poor and radiotherapy was discontinued after the multidisciplinary team consultation. Thus, the abdominal wall incision was treated with systemic chemotherapy with oxaliplatin and gemcitabine 1 month after the incision healed. At 3 months of follow-up after surgery, abdominal enhanced CT showed that the abdominal wall tumor had recurred and metastasized. Currently, the patient is undergoing the standard treatment for urachus adenocarcinoma.

## Discussion

The diagnosis of umbilical nodular invasive adenocarcinoma in the reported case was confirmed by the pathological consultation reports of two medical centers. Because the adenocarcinoma was non-specific, secondary metastatic cancer was initially suspected. After secondary metastasis had been ruled out in our medical center, the Sun Yat-sen University Cancer Center confirmed that this was a rare case of urachus adenocarcinoma, based on the location of the tumor and the relevant IHC findings. No similar medical cases have been reported in literature search.

In the reported patient, navel induration and pain occurred 3 months after surgery. However, given there was no history of malignant tumor diagnosis, the formation of foreign body granulomas or scar hyperplasia pain was assumed during outpatient follow-up and the patient was treated conservatively. At that time the possibility of a malignant mass was not considered. We believe that the key to diagnosing such cases is to improve the doctor’s understanding of a potential malignant mass in the incision. In our effort to determine the histological origin of the umbilical nodular adenocarcinoma, the patient’s medical history combined with CEA(+) and CA199(+) IHC findings indicated the digestive system as the potential source, especially cholangiocytes. Nevertheless, radiography and gastrointestinal endoscopy did not reveal any evidence of malignancy in the digestive system.

Laparoscopic puncture hole adenocarcinoma implantation metastasis is a rare complication of LC surgery [5]. When a patient with gallbladder cancer is clearly diagnosed before or after surgery and a lump appears in the postoperative incision, it can generally be considered that the incision tumor is implanted. However, in patients without a history of malignant tumors, induration or lumps in incisions occurring after surgery are more likely to be confused with scars, inflammation, and foreign body masses. In LC surgeries with non-cancerous pathological results of the gallbladder, the clinical manifestation of incision tumor implantation is mostly induration or mass in the puncture hole, which occurs 6–16 months (median 10 months) after surgery [5–7]. In our case, CT examination revealed a uniformly dense, high-density opacity of the puncture aperture [8]. This is related to the diagnosis rate of gallbladder cancer and pathological biopsy method [9]. At present, the pathological routine is to take two pieces of tissue at the neck, body, and edge of the gallbladder duct for paraffin embedding. If any microscopic tumor lesions are found in these biopsies, the number of sections will be appropriately increased to examine the entire gallbladder. Thus, cases of invasive cancer that are not obvious or are not detected in the initial pathological sections can be found in subsequent parts [10]. However, although local suspicion of gallbladder cancer appropriately increases the number of sections examined, there is still a theoretical possibility that gallbladder cancer may be missed.

After multiple imaging tests of the digestive system and pathology of the gallbladder were confirmed to be negative, we cooperated with the Department of Pathology of the Sun Yat-sen University Cancer Center. By observing the morphology of tumor cells, in combination with the tumor’s IHC profile and specific location, we determine the diagnosis to be urachus adenocarcinoma. The incidence of urachus cancer is low, accounting for approximately 0.01% of all adult malignant tumors and 0.2% of all bladder tumors. Its typical incidence age is 50–70 years, with a male-to-female ratio of approximately 1.8:1 [11]. The location of the growth site is connected to the clinical manifestations of urachal carcinoma: 90% of tumors located at the distal end of the urachus can press on the bladder and break through the bladder after hematuria; 6% of tumors located in the middle of the urachus or invading the abdominal wall can reach the mass in the lower abdomen; 4% of tumors located near the urachal tube ruptured early and the umbilical cord flowed out with mucous or bloody fluid [11]. The onset of urachus cancer is insidious, and due to its deep location next to the Retzius space there are generally no clinical manifestations in the early stages; thus, most of these tumors invade the bladder, and present with gross hematuria as their initial symptom [12]. Ultrasound is the most common clinical examination method, and acoustic images of urachus cancer mainly show an uneven hypoechoic mass with calcification, often protruding into the bladder cavity. CT examination is also an important method for identifying urachus cancer, which mostly manifests in the area of the urachus near the top of the bladder and anterior wall. Consistent with the long axis of the urachus, uniform or uneven tumor density, common calcification and low-density mucus area, and mild to moderate strengthening of arterial phases, intensification of tumors in the venous and excretory phases is more obvious and the sagittal position can better show the relationship between the urachus, bladder, and tumor [13, 14]. The characteristics of urachus carcinoma shown by magnetic resonance imaging are as follows: low signal on T1WI, slightly lower signal on T2WI, limited DWI spread and high signal, obvious enhancement of the solid part of the tumor, and no reinforcement of the cystic component on enhanced scanning [15]. The histological type of urachus carcinoma is mainly invasive adenocarcinoma [16, 17], followed by mucinous cell carcinoma, transitional cell carcinoma, clear cell carcinoma, and squamous cell carcinoma. IHC of urachus carcinoma is often positive for CK20, CK7, CD15, CDX2, Villin, CK34βE12, MLH1, MSH2, and MSH6 [18, 19].

In this case, the patient complained of pain in the umbilical nodule, but this was not accompanied with rupture and exudate from the skin nor did she have the usual symptoms of urachus carcinoma. Ultrasonography showed uneven hypoechoic nodules under the skin of the navel, with visible borders, no capsule, and no blood flow signal in the nodules. In addition, abdominal CT showed subcutaneous nodules in the navel with uniform density, no calcifications, and low-density liquid dark areas. However, the relevant preoperative examination did not suggest the possibility of malignancy. The history of LC surgery with induration of the incision after surgery further misled us to consider poor healing of the umbilical incision scar after surgery. However, even when nodular pathology suggested invasive adenocarcinoma, the patient was still considered to have a rare gallbladder-negative pathology and secondary puncture hole implantation metastasis. This mindset is more limited to one’s own professional framework, and does not consider the rare disease of urachal cancer. The diagnosis was finally determined to be urachus adenocarcinoma, and was achieved based on the tumor’s IHC profile, cell morphology, and specific location.

Transumbilical LC is widely practiced clinically as the gold standard for gallbladder disease. Measures to prevent incision implantation after LC surgery are as follows: 1) improving the diagnosis rate of gallbladder cancer and reducing the number of accidental gallbladder cancers. The primary cause of incision implantation after LC is accidental gallbladder cancer. The preoperative diagnosis rate of gallbladder cancer is very low, at only 10%. For older patients with a long medical history, when ultrasound and CT examination show irregular thickening of the gallbladder wall and gallbladder calcification the possibility of gallbladder cancer should be considered. In such cases, frozen pathological examination can be performed immediately during surgery to confirm the diagnosis. 2) Taking precautions during LC operation. The possibility of gallbladder cancer should be considered when preoperative and intraoperative findings suggest irregular thickening of the gallbladder wall, gallbladder calcification, etc. In such cases, a specimen bag should be used to minimize the spread of possible tumor cells. However, the routine use of a specimen bag as the new gold standard for all laparoscopic cholecystectomy specimens remains controversial [20]. When a gallbladder specimen is removed, gallbladder rupture and bile leakage should be avoided. Gallbladder or bile duct tumors that do not penetrate the serous membrane may increase the chance of incision implantation due to gallbladder rupture, tumor cells entering the free abdominal cavity, or touching the incision. At the same time, the incision should be protected to avoid air leakage. When air leaks, gas leaks around the cannula creating a chimney effect that has the potential to cause tumor cell accumulation at the incision site. Clinically, in patients with postoperative induration that does not improve after conservative treatment, rare abdominal wall malignant tumors should be considered. Preoperative needle biopsy may be the diagnostic method with the least trauma and greatest benefit for such patients.

## Conclusion

In conclusion, transumbilical LC is routinely performed clinically, and based on the findings of the reported case we advise caution. Specifically, when there is a persistent mass induration in the navel after LC surgery, the possibility of incision tumor should still be considered, rather than simply excluding the possibility of a malignant mass based on a non-cancer medical history. Also, understanding the rare possibility of urachus cancer is conducive to better guiding clinical work.

## Data Availability

The original contributions presented in the study are included in the article/Supplementary Material, further inquiries can be directed to the corresponding authors.
