# Supplementary material for: Urachus adenocarcinoma mistaken for umbilical incision implant cancer after laparoscopic cholecystectomy: a case report
Source: Pathol Oncol Res. 2023 Dec 22;29:1611334. doi: 10.3389/pore.2023.1611334 (PMC10766800; doi:10.3389/pore.2023.1611334)
Supplement: Supplementary file 1 [file DataSheet1.DOCX]

**Supplementary materials**

**Histopathology and immunohistochemistry**

All tissue samples were collected, fixed, embedded in paraffin, stained with hematoxylin and eosin (HE), and the tissue samples were stained for immunohistochemistry. CK7(FabGennix), CK20(RayBio), CEA(Abcam), CDX-2(Elabscience), CR(Abcam), TIF-1(Epigentek), CA199(SAB), SYN(Abcam), CgA(Abcam), D2-40(LBP), MC(Abcam), MUC5AC(Abmart), KI-67(Biosensis) staining were conducted according to the manufacturer's instructions.


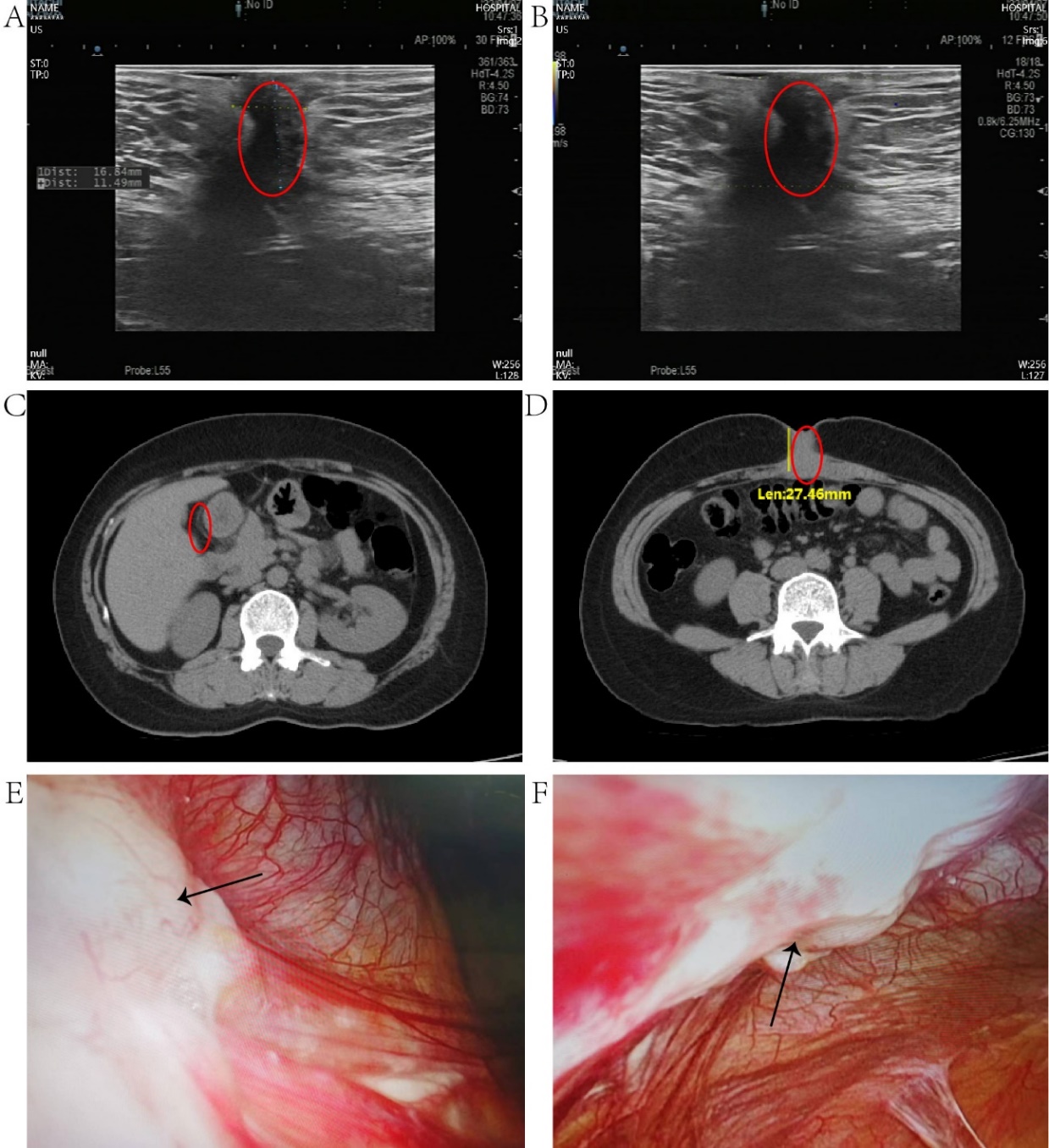


**FIGURE S1** Ultrasound and plain scan CT examination before the first umbilical nodule resection and intraoperative findings. A, B: Ultrasound examination before the first umbilical nodule resection; C, D: Plain scan CT examination before the first umbilical nodule resection; E, F: intraoperative findings


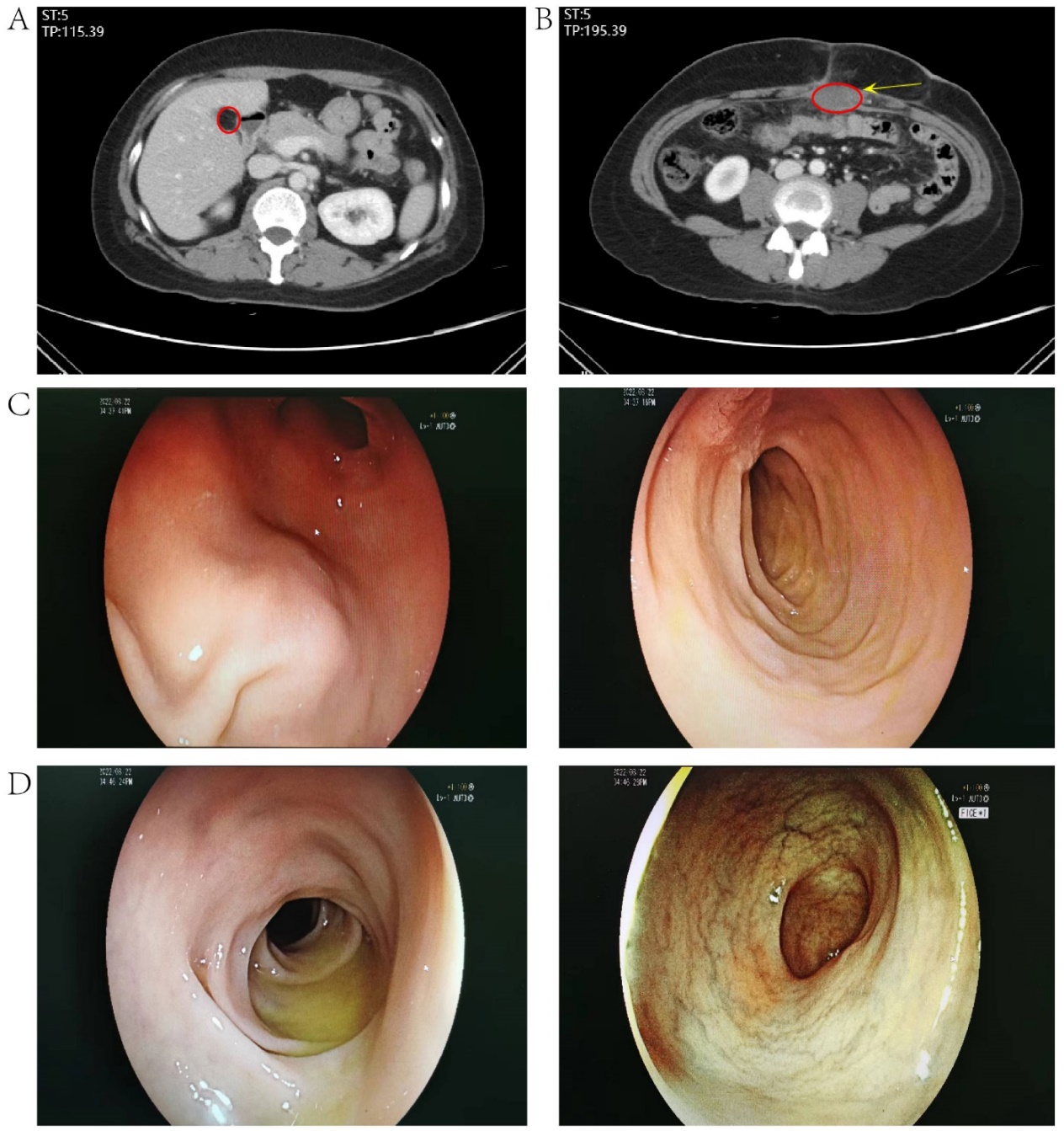


**FIGURE S2** Results of contrast-enhanced CT and gastrointestinal endoscopy after the first umbilical nodule resection. A, B: Contrast-enhanced CT examination after the first umbilical nodule resection; C: Gastroscopy examination after the first umbilical nodule resection; D: Colonoscopy examination after the first umbilical nodule resection.
